# Association of Acculturation and Latino Parents’ Oral Health Beliefs and Knowledge

**DOI:** 10.3390/children8030243

**Published:** 2021-03-22

**Authors:** Tamanna Tiwari, Anila Poravanthattil, Nayanjot Rai, Anne Wilson

**Affiliations:** 1 School of Dental Medicine, University of Colorado, Aurora, CO 80045, USA;; 2 Children’s Hospital, University of Colorado, Aurora, CO 80045, USA;

**Keywords:** acculturation, health beliefs, oral health, barriers to care

## Abstract

The purpose of our study was to explore the association of acculturation and Latino parent behavioral and psychosocial characteristics. A cross-sectional survey was conducted with 197 parent-children triads. Participating parents completed survey questions encompassing oral health knowledge, behaviors and beliefs from a validated oral health instrument. The mean score for acculturation in this sample was 3.8, where acculturation was dichotomized to a categorical variable. The bivariate associations between the independent variables (caregiver psychosocial factors and socio-economic factors (SES) factors) and acculturation (more/less acculturated) were conducted using logistic regression analysis, and for the final model a multivariate logistic regression model was used. In the bivariate analyses, less acculturated parents reported lower oral health knowledge (*p* = 0.02), higher social support (*p* = 0.028) and chronic stress (*p* = 0.015) and lower perceived susceptibility to dental caries in their children (*p* = 0.039). The bivariate analysis demonstrated that less acculturated parents had less education and employment (*p* < 0.0001) than more acculturated parents. The multivariate logistic model demonstrated that social support (*p* = 0.028), chronic stress (*p* = 0.015) and health beliefs as barriers to access dental care (*p* = 0.039) were higher in less acculturated parents compared to more acculturated parents. Less acculturated parents demonstrated lower oral health knowledge, higher stress and more barriers to accessing oral health care for their children. Oral health interventions for Latino families should incorporate strategies that include consideration of parental oral health beliefs.

## 1. Introduction

“Acculturation is defined as the process by which individuals from a community may adopt the values and behaviors from another culture, and this may, in turn, affect their beliefs” [1–3]. The adopted ideas and changes in the cultural norms and practices of an immigrant community may influence their healthcare-seeking and prevention behaviors, which ultimately may affect their health outcomes [4]. Acculturation can be an essential factor in maintaining the health of immigrant children, and in particular, Latino children [4].

Immigrant children account for 25% of all children in the United States [5]. In the United States, 18.7 million children are immigrants, of whom 55% are Latino [5]. Latino children experience the poorest oral health outcomes, especially with regard to dental caries, of all populations in the US, with the exception of American Indian children [6]. The high prevalence of dental caries in Latino children compels us to think about the underlying causes of the oral health disparities faced by this population. Social determinants of oral health are the root causes of such disparities, and a major determinant for Latino children is parental acculturation [7].

Few oral health outcomes, such as those associated with oral health behaviors including oral hygiene, dental visits and dental service utilization, have been studied in relation to the level of parental acculturation [8–15]. In a cross-sectional study that used language as a proxy measure of acculturation, more acculturated or English-speaking Latino mothers had a higher inclination to use dental services for the prevention of dental caries and reported higher self-efficacy related to taking their children for preventive visits and maintaining their child’s oral hygiene [13]. In addition, their children experienced lower rates of dental caries compared to children of less acculturated mothers. In another study conducted with Latino mothers, the level of acculturation measured by length of stay in the USA was one of strongest predictors of high caries prevalence in children [14]. A study that analyzed household acculturation using the measure of language and parent nativity from the National Survey of Children’s Health concluded that, as the level of acculturation increased in the household, Latino children’s oral health also improved [8].

These studies demonstrate some evidence of a relationship between acculturation and parental beliefs. However, most studies use proxy measures to measure acculturation, and few analyze the parental characteristics, such as behaviors or attitudes, that are influenced by acculturation [16]. This study helps to fill that gap by applying a validated measure of acculturation that includes several psychosocial, behavioral and demographic parental characteristics. The purpose of our study was to explore the association of acculturation and Latino parents’ oral health behavioral and psychosocial factors.

## 2. Materials and Methods

### 2.1. Study Design and Sample Size

A cross-sectional survey was conducted with 200 Latino parent-child triads, enrolled in health centers in the Denver Metro area in Colorado. Each enrolled triad consisted of a Latino parent that was at least 18 years of age with two children, of which one child was between birth and 6 years of age and the second child was between birth and 10 years of age. The study enrollment started in July 2017 and was completed in March 2019. This study was approved by the Colorado Multiple Institutional Review Board (COMIRB).

### 2.2. Data Collection

#### 2.2.1. Basic Research Factors Questionnaire

The questionnaire used in this study was a partial selection of items from the Basic Research Factors Questionnaire (BRFQ). The BRFQ was developed to enhance understanding of parental influences on children’s oral health. The BRFQ is a 190-item questionnaire that surveys information relative to parents’ and children’s sociodemographic characteristics, parental oral health knowledge and behaviors, parental oral health attitudes, household characteristics, and health status. It is available in English and several other languages.

The parents were prescreened using the electronic health records for their children. The parent was approached by a member of the research team in the waiting area of the clinics to explain the study procedure. Spanish speaking parents were provided information about the study by certified translators. Parents who agreed to participate in the study signed the consent form prior to completing the questionnaire. Parents were asked to keep one of the children in mind while completing the survey, and that child was considered the primary child in the study. The survey was completed in English or Spanish, based on the parents’ preferred language, using an iPad and REDCap (Research Electronic Data Capture) electronic data capture tool hosted at the University of Colorado Anschutz Campus. REDCap is a secure, web-based software supporting clinical research data capture.

#### 2.2.2. Variables

##### Oral health behavior:

This is a twelve-item scale that collects an overall behavior score.

##### Oral health knowledge:

This is a fourteen-item scale that assesses parental knowledge related to oral hygiene routines and feeding practices.

##### Dental utilization knowledge:

Parent knowledge on utilization of oral health services is measured using five items.

##### Self-efficacy:

Self-efficacy measures parental confidence in the ability to engage in oral health behaviors that are recommended by the dentist for their child. All 10 items in this survey ask parents about their confidence level. The average of the responses was calculated for each participant, with larger numbers representing a greater degree of self-efficacy.

##### Health belief model:

Four concepts of the health belief model are included: perceived susceptibility, perceived seriousness, perceived benefits and perceived barriers. Responses to all items ranged from one to five (strongly disagree-strongly agree). The average of the items associated with each construct was computed. Items for perceived barriers were reverse-coded.

##### Multidimensional oral health locus of control:

Nine items assessed the multidimensional locus of control. The three subscales include internal locus of control, external locus of control—powerful others, and external locus of control—chance.

##### Parent stress index:

This scale measures perceived stress related to the caregiving role of the parents and includes nine items.

##### Social support:

This four-item measure evaluates the degree to which parents believe they have others available to help when needed.

Demographics include parents’ age, education, employment status and household size, the number of minors in the household, the number of years the family has lived at the current residence, and health insurance.

##### Acculturation:

Acculturation was the outcome variable in this study. The 12-item Acculturation Rating Scale for Mexican Americans (ARSMA-II) was used to measure acculturation.

### 2.3. Statistical Analysis

For descriptive analysis, the continuous variables were summarized with means and standard deviations (Table 1), and the categorical variables were summarized with counts and percentages (Table 2). Two tailed *t*-tests were conducted to test for differences in the means of predictor continuous variables (listed in Table 1) between the less and more acculturated groups. Chi-square testing was conducted to test for significant associations between the categorical parent health and health care access variables listed in Table 2 and the outcome variable.

Acculturation was dichotomized into more acculturated (score ≥ 3.8) and less acculturated (score < 3.8) caregivers, based on the 3.8 mean acculturation score cut-off. The bivariate associations between the independent variables (caregiver psychosocial factors and SES factors) and acculturation (more/less acculturated) were conducted using logistic regression analysis.

Variables were included in this full model only if they achieved a significance level of *p* ≤ 0.20 in bivariate analysis. Estimates and *p*-values were reported. All the data cleaning and analysis were conducted using SAS v9.4.

## 3. Results

Two hundred parent-child triads were enrolled in the study. The response rate of the invited parents was 98.5% and survey data was analyzed for 197 parents. The mean acculturation score for the sample was 3.8. For further analysis, acculturation was dichotomized into more acculturated (score ≥ 3.8) and less acculturated caregivers (score < 3.8), based on the 3.8 mean acculturation score cut-off. Table 1 shows the results for the *t*-tests. Significant differences were found in the means of three subscales of the health belief model—perceived severity (mean 3.07 vs. 2.78, *p* = 0.026), perceived barriers (mean 3.23 vs. 2.94, *p* = 0.015) and perceived susceptibility (mean 3.61 vs. 3.08, *p* = 0.001).

More acculturated parents reported significantly better general health (64.47%, *p* < 0.0001), better oral health (41.62%, *p* < 0.0001), better oral health literacy (74.62%, *p* < 0.0001), visiting the dentist more often (59.39%, *p* < 0.0001) and better oral health behaviors of brushing at least two times or more daily (61.42%, *p* < 0.0001). More acculturated parents also reported having dental insurance (75.63%, *p* < 0.0001), experienced less time to reach a dentist for preventive visits (69.54%, *p* = 0.044), had more frequent access to a car (78.68%, *p* < 0.0001), and missed work less frequently (75.63%, *p* < 0.0001) as compared to less acculturated parents.

Table 2 demonstrates the results of the bivariate logistic regression associations between independent predictor variables and acculturation. It was found that less acculturated parents had significantly less oral health knowledge (E = −0.0241, *p* = 0.021), higher chronic stress (E = 1.2235, *p* = 0.027) and more social support (E = 2.0659, *p* = 0.009) as compared to more acculturated parents. They perceived more barriers to accessing dental care (E = −0.5523, *p* = 0.012) and perceived their children to be less susceptible to caries (E = −0.2848, *p* = 0.001) compared to more acculturated parents. In addition, less acculturated parents had significantly less educational attainment (E = −1.7216, *p* < 0.0001), were less frequently employed (E = −1.3963, *p* = 0.0009), and spent fewer years at their current residence (E = −1.7000, *p* = 0.001).

For the final model, all the potential variables (listed in Table 3) having a bivariate association with acculturation of *p* < 0.20 were included in a multivariate logistic regression model. Only three variables had significant association with the outcome variable: social support, chronic stress, and one subscale of the health belief model—perceived barriers (Table 3 results indicate that social support (E = 2.1539, *p* = 0.028), chronic stress (E = 1.7161, *p* = 0.015) and barriers to access dental care (E = −1.3076, *p* = 0.039) were higher in less acculturated parents compared to more acculturated parents.

## 4. Discussion

Acculturation is an important social determinant of oral health for immigrant communities [4,16]. This study demonstrated that acculturation was associated with parental oral health attitudes, beliefs and behaviors. Less acculturated parents had low oral health knowledge, perceived more barriers to accessing the dentist for preventative dental care, perceived their children to be less susceptible to dental caries and reported higher chronic stress. These findings are consistent with the literature. On the other hand, it was noteworthy that parents with lower acculturation exhibited higher levels of social support.

In addition, this study provided insight relative to the oral health knowledge, behavior and belief characteristics of more acculturated parents. As anticipated, more acculturated parents reported better oral health and general health, higher income and education, dental insurance and more frequent access to a car, compared to less acculturated parents. These social determinants of health have been associated with better oral health outcomes in Latino children in previous studies [17–19].

Less acculturated parents perceived greater barriers in maintaining preventive dental visits for their children. In a previous study conducted with the Latino population from the same dental center, it was demonstrated that less acculturated mothers perceived more barriers to oral health behavioral adherence. A significant correlation was seen for knowledge of dental utilization and perceived barriers. As dental utilization knowledge improved for less acculturated participants, they perceived greater benefits from adherent oral health behavior [13]. The literature iterates that acculturation is a continuous process, and as immigrant families are exposed to the new culture, their behaviors, beliefs and knowledge evolve over time. Longitudinal research is warranted to study the changing relationships of acculturation with oral health attitudes and other psychosocial factors.

Less acculturated parents also had higher levels of chronic stress; it has been shown in earlier studies that immigrants are affected by multifactorial post-migration stress factors, such as assimilating in an unfamiliar country and culture [20]. Chronic stress can also have an effect on the development of oral disease [21].

A noteworthy finding seen in this study is that less acculturated parents had higher social support. This was also reported in the primary publication from this study, which reported data from the in-depth interviews [22]. Parents gave a detailed account of the support they received from family and friends in the caretaking of their children, including support in dental visits and transportation [22]. A recent study that examined a large dataset from the Hispanic Community Heath Study/Study of Latinos reported that social support was protective against dental caries [23]. The influence of social support systems, communal relationships, and neighborhood environments has been known to have a positive impact on various health domains, especially in the medical field, including recovery from illness, health maintenance, chronic illness self-management, and medication adherence [24]. The literature shows that Latinos have strong family-centered social support networks. The reliance on extended kin for support in decision-making is termed “familism” [25] and is considered a core value for Latino communities, although this is inversely related to increased acculturation and/or higher socioeconomic status [26]. Some studies propose a protective effect of specific ethnocentric values in Latino communities, like reciprocity and social relationships, which tends to decrease as the length of residence in the country of immigration increases [27].

Limitations of the study include recruitment of participants from an existing group of patients at a pediatric dental practice in an urban community. Thus, results may not be transferrable to other communities that are non-urban or to children without a dental home. Given that the study was cross-sectional, conclusions about causal relationships are not possible. Strengths of the study include that the behavior, knowledge and health belief survey items were validated in this population [13]. Other strengths are that the study sample was adequately powered and adds to the literature, as there is a paucity of literature on Latino acculturation and assessment of oral health outcomes and behavior, knowledge and beliefs.

## 5. Conclusions

In conclusion, this study provides evidence of the impact of acculturation on oral health beliefs in Latino parents. It puts a spotlight on how perceived health beliefs on barriers to care and stress are associated with levels of acculturation. Furthermore, social support is an important component of Latino communities and is influenced by acculturation. Oral health care providers and researchers can apply this information to inform oral health interventions aimed at promoting oral health in Latino families. Therefore, it is recommended to address acculturation in oral health preventive efforts.

## Figures and Tables

**Table 1.** Difference in means of less and more acculturated parental psychosocial factors. (Cut-off = 3.8).

Parental Psychosocial Factors	More Acculturated Percent (%) Mean (SD)	Less Acculturated Percent (%) Mean (SD)	*p*-Value
Overall behavior	61.07%	61.12%	0.878
Oral health behavior—hygiene	61.59%	59.81%	0.718
Oral health behavior—diet	75.0%	76.60%	0.828
Oral health knowledge	73.32%	69.53%	0.151
Social support	1.16 (0.31)	1.19 (0.33)	0.499
Parent stress index	2.53 (1.003)	2.34 (0.69)	0.481
Chronic stress	1.77 (0.58)	1.72 (0.49)	0.820
Self-efficacy	2.53 (0.49)	2.60 (0.47)	0.529
Knowledge on dental utilization	3.68 (0.98)	3.45 (1.08)	0.113
LOC Internal	4.10 (0.87)	3.93 (1.08)	0.154
LOC External—others	2.34 (1.07)	2.32 (1.02)	0.909
LOC External—chance	2.75 (1.22)	2.48 (1.15)	0.254
HBM Severity	3.07 (1.06)	2.79 (0.93)	0.026
HBM Barriers	3.23 (0.91) *	2.94 (1.02)	0.015
HBM Susceptibility	3.61 (0.91)	3.08 (1.16)	0.001
HBM Benefits	4.11 (1.17)	3.84 (1.81)	0.206

* reverse coded. SD = standard deviation, LOC= locus of control, HBM= health belief model.

**Table 2.** Bivariate association between acculturation (less acculturated/more acculturated) and parental factors.

Parental Psychosocial Factors	Estimate	* *p*-Value
Oral health behavior	−0.00127	0.926
Oral health knowledge	−0.0241	0.021
Social support	2.0659	0.009
Parent stress index	0.2855	0.328
Chronic stress	1.2235	0.027
Self-efficacy	−0.0923	0.831
Knowledge on dental utilization	−0.3526	0.093
LOC Internal	−0.2791	0.179
LOC External—others	0.2949	0.159
LOC External—chance	0.2017	0.263
HBM Perceived Severity	−0.1958	0.371
HBM Perceived Barriers	−0.5523	0.012
HBM Perceived Susceptibility	−0.2848	0.001
HBM Perceived Benefits	−0.1627	0.357
**Parental SES factors**		
Parents’ Education	−1.7216	<0.0001
Parents’ Employment	−1.3963	0.0009
Household Size	0.0241	0.884
Household Minors	0.0396	0.847
Years in Household	1.7000	0.001

* logistic regression model.

**Table 3.** Final model for association of acculturation with significant parental factors, adjusting for parental SES. Outcome: acculturation (more/less) (*p* < 0.20).

Parental Psychosocial Factors	Estimate	*p*-Value
Oral health knowledge	0.00251	0.938
Social support	2.1539	0.028
Chronic stress	1.7161	0.015
Knowledge on dental utilization	−0.3019	0.496
LOC—internal	0.7944	0.144
LOC—powerful others	0.4234	0.289
HBM Perceived Barriers	−1.3076	0.039
HBM Perceived Susceptibility	0.4834	0.283
Parents’ education	0.1073	0.892
Parents’ employment	−0.0490	0.956
Years in household	−2.4542	0.077

The overall *p*-value for this model is 0.032.
